# Effectiveness of a Technology-Based Supportive Educational Parenting Program on Parental Outcomes in Singapore: Protocol for a Randomized Controlled Trial

**DOI:** 10.2196/resprot.8062

**Published:** 2018-01-10

**Authors:** Shefaly Shorey, Yvonne Peng Mei Ng, An Ling Siew, Joanne Yoong, Evalotte Mörelius

**Affiliations:** ^1^ National University of Singapore Singapore Singapore; ^2^ National University Hospital Singapore Singapore; ^3^ Linkoping University Norrkoping Sweden

**Keywords:** parents, satisfaction, perinatal, self-efficacy, social support

## Abstract

**Background:**

Supportive educational programs during the perinatal period are scarce in Singapore. There is no continuity of care available in terms of support from community care nurses in Singapore. Parents are left on their own most of the time, which results in a stressful transition to parenthood. There is a need for easily accessible technology-based educational programs that can support parents during this crucial perinatal period.

**Objective:**

The aim of this study was to describe the study protocol of a randomized controlled trial on a technology-based supportive educational parenting program.

**Methods:**

A randomized controlled two-group pretest and repeated posttest experimental design will be used. The study will recruit 118 parents (59 couples) from the antenatal clinics of a tertiary public hospital in Singapore. Eligible parents will be randomly allocated to receive either the supportive educational parenting program or routine perinatal care from the hospital. Outcome measures include parenting self-efficacy, parental bonding, postnatal depression, social support, parenting satisfaction, and cost evaluation. Data will be collected at the antenatal period, immediate postnatal period, and at 1 month and 3 months post childbirth.

**Results:**

Recruitment of the study participants commenced in December 2016 and is still ongoing. Data collection is projected to finish within 12 months, by December 2017.

**Conclusions:**

This study will identify a potentially clinically useful, effective, and cost-effective supportive educational parenting program to improve parental self-efficacy and bonding in newborn care, which will then improve parents’ social support–seeking behaviors, emotional well-being, and satisfaction with parenting. It is hoped that better supported and satisfied parents will consider having more children, which may in turn influence Singapore’s ailing birth rate.

**Trial Registration:**

International Standard Randomized Controlled Trial Number (ISRCTN): 48536064; https://www.isrctn.com/ISRCTN48536064 (Archived by WebCite at http://www.webcitation.org/6wMuEysiO)

## Introduction

### Background

The addition of a newborn to a family unit necessitates an environment of change and uncertainty [1] as parents are confronted with various new challenges and struggle to adapt to the new dynamics at home [2]. The parenthood experience is largely influenced by many factors including parents’ confidence levels, emotional well-being, social networks, and the quality of their support system [3]. Such environmental factors are capable of acting as a security blanket to buffer against the stressors that comes with parenthood, or add on to the stress parents are already facing, and such negative experiences can deter couples from becoming parents again. It is crucial to maintain positive emotional well-being in parents as studies have shown that maternal psychosocial stress can influence dynamics in the family [4-6].

In view of the dipping fertility rate [7,8], the Singapore government has been coming up with attractive incentive packages [9-11]; however, these have proven to be futile in boosting fertility rates. Low fertility rates are associated with multiple factors including an economic drain [12], parental expectations of childbirth, and previous negative birth experiences [13,14]. Previous studies have found that perinatal experiences were one of the major factors affecting birth rate [15,16]. Negative perinatal experiences such as a lack of support during this crucial period may influence a parent’s decision to give birth again [17]. In Singapore, perinatal support is mainly provided by maternity hospitals [18]. Unlike certain parts of Western Europe, continuity of care post hospital discharge by community care nurses is lacking in Singapore [19].

To assist parents in transiting smoothly into parenthood, antenatal classes and postnatal educational sessions are available at the hospitals in Singapore [20]. However, because of early discharges [21] and having so much to teach, parents are overloaded with too much information [22], which limits the effectiveness of these educational sessions. In a local study, mothers found that informational support provided by the hospital was unsatisfactory [20] as the most crucial period for parents is usually the first few weeks post discharge, when parents are left to handle stressful situations on their own. Moreover, current perinatal services in Singapore focus much of their attention on supporting women’s transition to early motherhood during hospitalization and pay less attention to fathers. A study conducted in Canada revealed that fathers did not feel supported by the hospital environment with regard to promoting their involvement with their newborn [23]. Therefore, there is a need for a theory-based educational program that focuses on facilitating smooth transition to parenthood for both parents.

Previous local studies were mainly focused on first-time mothers [24] or the early postpartum period [25]. There is a limited amount of educational programs that are developed for both first-time and experienced parents across the perinatal period. Support for parents from the antenatal period until the postnatal period is recommended in both international [26-28] and local [25] studies. Additionally, most of the available educational programs are delivered face-to-face, in which information is relayed didactically [27,29]. The shortage of midwives and nurses limits the feasibility of such programs as they are not cost-effective [29].

With the advancement in technology, the use of technology-based educational programs with new parents during the postnatal period both internationally [22,30,31] and in local context [25] have found to be successful in influencing parental outcomes in the postnatal period. However, because of the lack of a rigorous experimental study design [22,31] and limited postnatal focus [25,30,32], there is a need to develop an intensive family-oriented perinatal educational program that is professionally based and provided at a low cost, utilizing technology to enhance accessibility to all parents without compromising its effectiveness on parental outcomes. As such, this study aims to develop a technology-based supportive educational parenting program (SEPP) to support both fathers and mothers across the perinatal period by targeting different variables through the different components of the SEPP.

### Theoretical Framework

**Figure 1:**
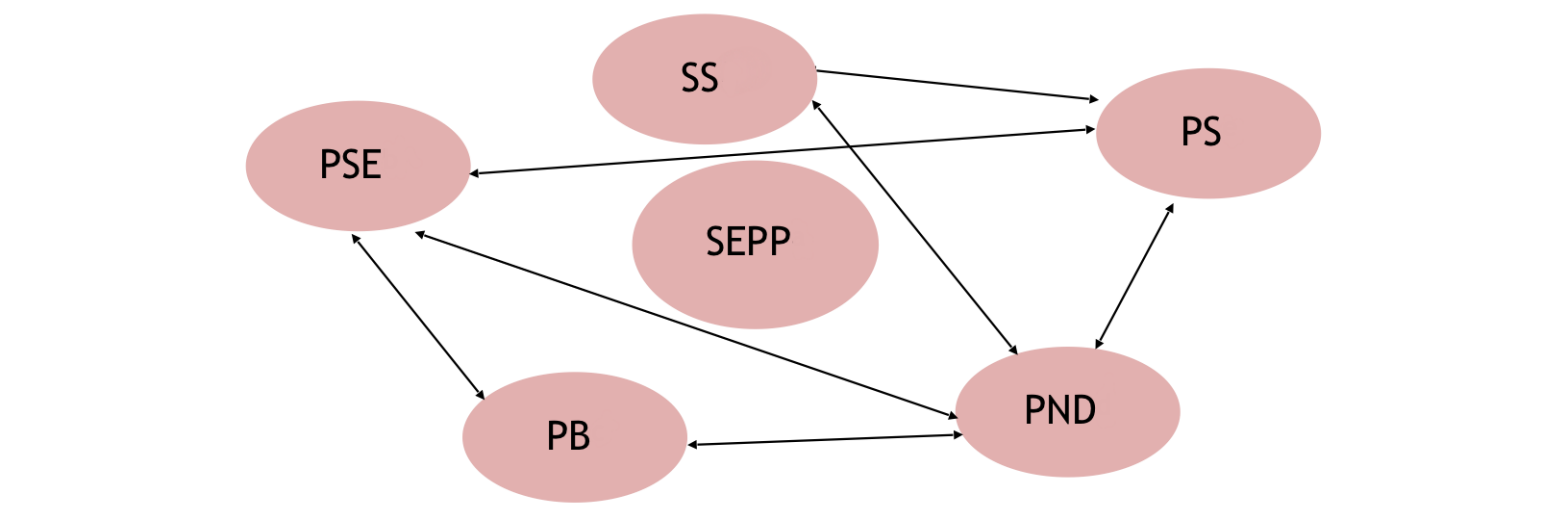
Theoretical Framework showing interrelationship between outcome variables. SEEP: Supportive educational parenting programme; PSE: Parenting self-efficacy; PB: Parental bonding; PND: Postnatal depression; PS: Parenting satisfaction; SS: Social support.

The theoretical framework guiding the SEPP is Bandura’s self-efficacy theory and Bowlby’s attachment theory [32,33]. According to Bandura’s self-efficacy theory [33], parenting self-efficacy (PSE) is considered one of the major determinants of competent parenting and the well-being of a newborn and is related to parental emotional well-being such as postnatal depression and the social support available for parents. Bowlby also theorizes that early parent-to-infant attachment provides the foundation of psychological well-being and, later, the development for infants in building social relationships, which can be influenced by PSE, emotional well-being, and social support [32]. Social support is associated with high PSE, which is in turn related to better emotional well-being and enhanced parental bonding with their newborn [24,27,34,35], which may ultimately lead to better parenting satisfaction. Hence, PSE, parental bonding, emotional well-being, and social support are interrelated variables, and the SEPP aims to target these variables to improve parental outcomes. This theoretical framework is represented in Figure 1.

### Aims

The aims of the study were to (1) examine the effectiveness of the SEPP on parental outcomes, including PSE (primary outcome), parental bonding, postnatal depression and anxiety, social support, and parenting satisfaction; (2) evaluate the cost-effectiveness of the SEPP compared with routine perinatal care; and (3) process evaluation of the SEPP by assessing its strengths and weaknesses from parents’ perspectives.

### Hypotheses

The hypotheses are (1) When compared with those in the control group receiving routine care, parents receiving the SEPP will report significantly (a) higher levels of self-efficacy in newborn care, (b) higher levels of parental bonding, (c) lower levels of depression and anxiety, (d) higher levels of social support received, and (e) higher levels of parenting satisfaction and (2) It is more cost-effective to provide the SEPP than routine care.

## Methods

### Design

A randomized controlled two-group pretest and repeated posttest experimental design will be used. Couples (n=118 participants) recruited from a tertiary hospital will be randomly assigned into the two groups (intervention group receiving the SEPP and routine care or control group receiving only routine care). Data will be collected via questionnaire surveys using locally validated and reliable instruments, medical reviews, semistructured face-to-face interviews, and telephone interviews.

### Components of the Supportive Educational Parenting Program

Parents who are assigned to the intervention group will receive the SEPP and routine perinatal care provided by the hospital. Parents in the control group will receive only routine care. The routine care includes antenatal check-ups with an obstetrician and short postnatal stays in the hospital. During antenatal check-ups, the progress of the pregnancy is assessed via maternal bio-physiological profile, height and weight, and abdominal palpation. Antenatal educational classes are available; however, they are charged and not fully utilized by parents because of their lack of awareness [24]. During postnatal stay, parents will receive care from an obstetrician, nurses, and lactation consultants. Parent-craft educational classes are available. However, because of the short hospital stays, most of the parents are too tired to attend these sessions [24]. The SEPP consists of three main components, as presented in Table 1.

In Singapore, the mobile phone penetration rate was approximately 98% in 2016 [36], which is the highest globally. Moreover, under the smart nation initiative, Singapore is moving toward delivering holistic health care to the population through technological innovations [37]. Hence, delivering the SEPP in the form of a mobile health (mHealth) app is not only feasible but also a sustainable and convenient form of education delivery for health care professionals (HCPs) and parents. All educational contents in the app has been reviewed and approved by the ethics board.

**Table 1:** Details of the Supportive Parenting Educational Program.

No.^a^	Mode and period of delivery	Approximate duration of the session	Topics covered
1	Telephone-based antenatal educational session	30 min	Parental self-efficacy and bonding (eg, definitions of parental self-efficacy and bonding, why are they important, and how they can be enhanced Expectations in the immediate postpartum period Emotional needs of parents both during pregnancy and after the child’s birth
2	Telephone-based immediate postnatal educational session	60 min	Reinforcement on the topics covered during antenatal period, including parenting self-efficacy, parental bonding, and postnatal depression Coverage on parent-craft class topics including baby bathing and breastfeeding Role of fathers during postnatal period
3	mHealth^b^ app-based follow-up educational session	1 month access to the mHealth app	A knowledge-based content on topics including newborn and maternal care Audio recordings of the knowledge-based content Videos on various newborn care tasks such as baby bathing and breastfeeding demonstrated by a trained midwife using a live baby. Each video is approximately 8 min long on average A discussion forum Push notifications

^a^Number in series.

^b^mHealth: mobile health.

**Figure 2:**
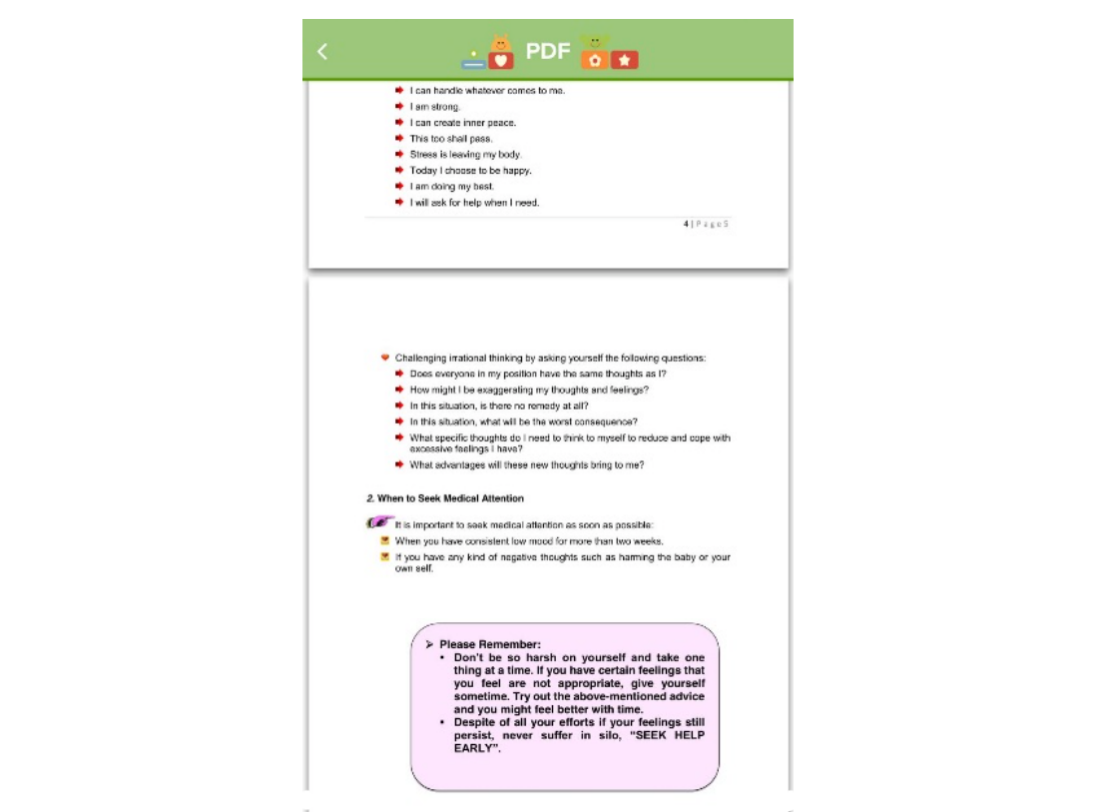
Screenshot of knowledge-based content.

**Figure 3:**
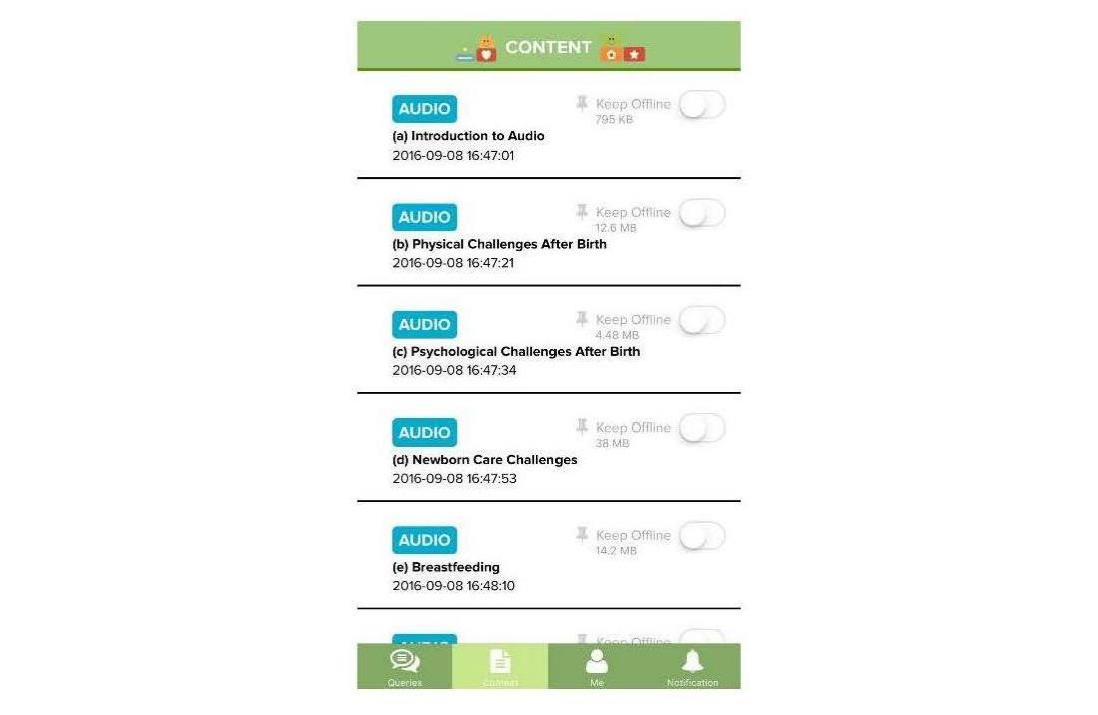
Screenshot of audio recordings.

**Figure 4:**
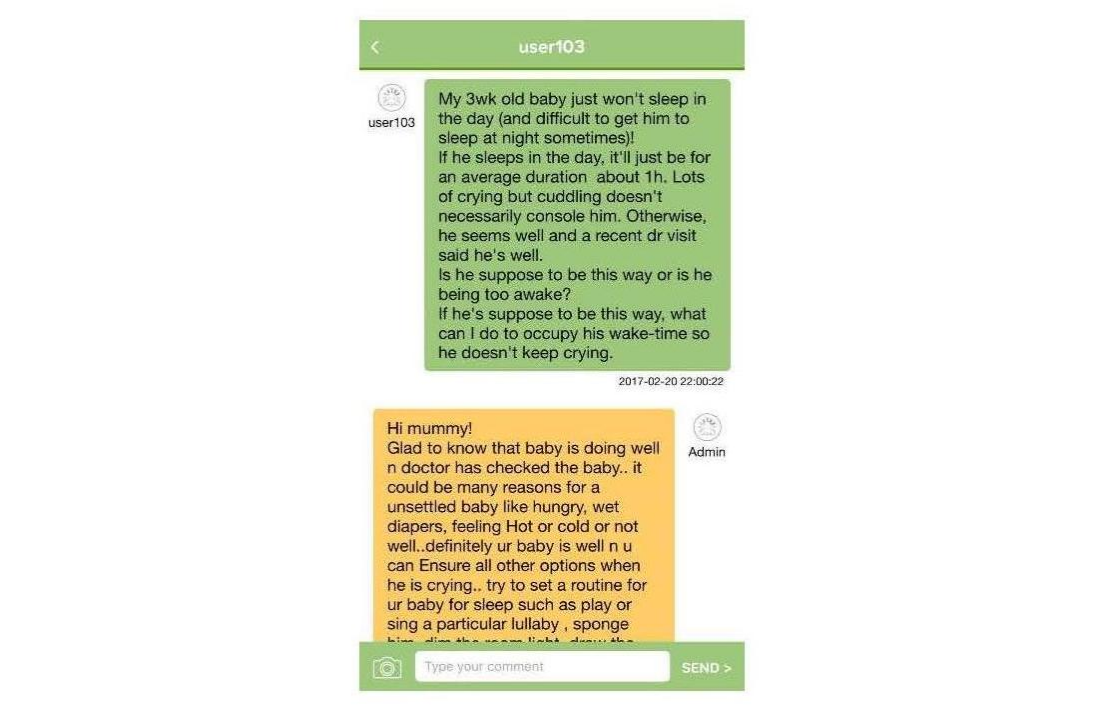
Screenshot of discussion forum.

**Figure 5:**
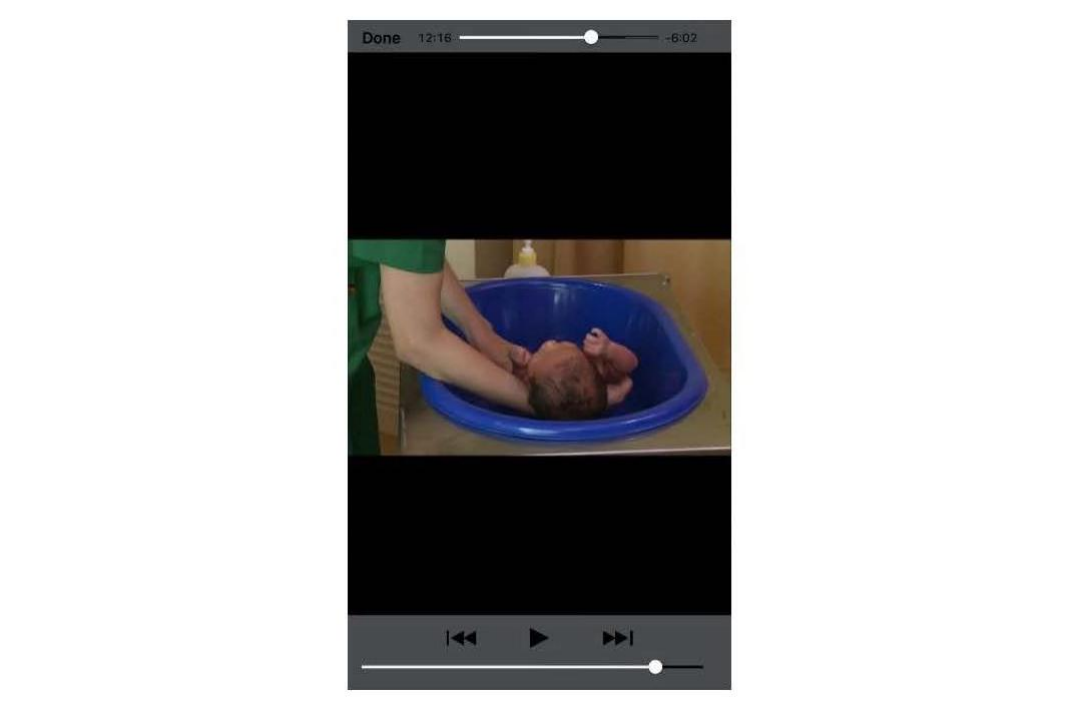
Screenshot of videos.

**Figure 6:**
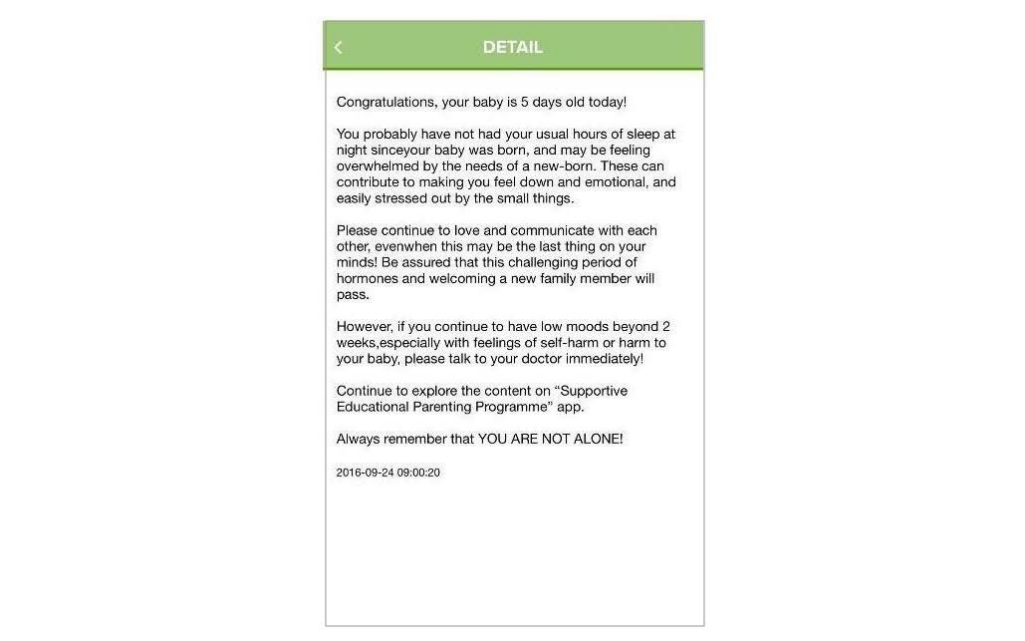
Screenshot of push notifications.

The knowledge-based content in the mHealth app includes evidence-based information on ways to enhance parental self-efficacy and bonding, newborn care and maternal self-care tasks, emotional challenges and ways of dealing with it, breastfeeding-related information, and insights for new parents for a smooth transition to parenthood. The discussion forum will be a platform for parents to ask any questions they might have. All parental queries will be answered by a trained midwife (research assistant 1, RA1) once a day for the first 4 weeks post childbirth because this period is the most stressful period for new parents in the early post-partum period [38,39]. Additionally, other parents can also provide insights to these queries by sharing their personal experiences. According to Bandura’s self-efficacy theory [33], self-efficacy and a help-seeking behavior (social support) can be enhanced when tasks are learned from others in a similar situation; therefore, parents are encouraged to interact with other parents in the discussion forums. Daily push notifications regarding important milestones on parenting will be sent to users. Each notification appears on the user’s home screen, which aims to pique the user’s interest and serves as a reminder to continue exploring the rest of the content-rich app. Participants’ access to the mHealth app will be monitored (by adding the function of time spent in accessing materials) to record the dosage of the intervention each participants receives. The app was developed by an external vendor who is an expert in mHealth app development. The research team and the external app developer have been keeping in close contact for the development of the app. Screenshots of the features of the app as examples are shown in Figures 2-6.

To ensure consistency, the same RA (RA1) will deliver the telephone-based prenatal and postnatal educational component in the SEPP to all the participants in the intervention group. The protocol for the SEPP has been validated by an expert panel consisting of five HCPs (one nursing professor who is experienced in postnatal educational programs, one senior consultant-neonatologist, one senior consultant-obstetrician, and two experienced midwives in perinatal education) and two sets of parents.

**Inclusion criteria.**

1. Are 21 years and above
2. Are able to read and speak English
3. Have low-risk singleton pregnancy with more than 28 weeks of gestation
4. Have a mobile phone with Internet access
5. Plan to stay in Singapore for the first 3 months post delivery

**Exclusion criteria.**

1. Have self-reported physical or mental disorders that will interfere with their ability to participate in the study
2. Have high-risk pregnancy, including placenta-previa major, preeclampsia, and pregnancy-induced hypertension
3. Have complicated assisted delivery such as vacuum or forceps with 4th degree perineal tear of the mother
4. Gave birth to a stillborn or a newborn with congenital abnormalities or medical complications, or
5. Are a single parent

### Participants

The participants recruited will be couples (a father and mother dyad is considered one couple). The inclusion and exclusion criteria for participants who are mothers are shown in Textboxes 1 and 2.

The inclusion criteria for fathers are those who (1) are 21 years and above, (2) are able to read and speak English, (3) have a mobile phone with Internet access, and (4) plan to stay in Singapore for the first 3 months post delivery. The exclusion criteria for fathers are those who have self-reported physical or mental disorders that will interfere with their ability to participate in the study and/or are a single parent.

All couples are recruited antenatally and will be told beforehand that they may be excluded from the study if mothers are to experience complication during their pregnancy and the delivery of their newborn.

### Sample Size Determination

On the basis of previous studies, psychosocial and educational interventions in the perinatal period generally produce a medium size effect on outcome variables [27,28]. Hence, the SEPP intervention can also be assumed to have a medium effect size on outcome variables. A repeated measure analysis of covariance (ANCOVA) will be used to test for differences between two groups (intervention and control) and within four separate time points and for interaction (group x time) effects. The required sample size for the detection of a medium effect size of 0.3 (analysis of variance [ANOVA] *F* value) at a power of 80% and a significance level of 5% (two-sided) is 45 in each group [40]. Considering a 30.0% (30/100) attrition rate based on previous studies [27,29], a minimum of 118 participants (45x2 + 45x2x30%=90 + 28=118), with 59 in each group, is needed. A purposive sample of approximately 16 participants will be needed for the process evaluation. Parents with differing mean parenting efficacy (primary outcome) scores from both the intervention and control groups (8 from each group) will be selected to participate in the face-to-face interviews, until data saturation is attained.

### Randomization

The research randomizer will be used to randomly generate one set of 59 numbers ranging from 1 to 118. Generated numbers are assigned to the intervention group and the remaining to the control group. All 118 numbers will be placed in an opaque envelope. The principle investigator who will not be involved in the recruitment will hold the random numbers and pass it to RA1 for recruitment. After assessing eligibility and obtaining informed consent, the participants will be asked to pick a number from the opaque envelope, in which the number picked will determine the groups assigned.

### Outcome Measures and Instruments

Patients’ demographic data (eg, age, gender, ethnicity, and education) will be collected. In addition, the following instruments will be used to measure parental outcomes:

1. Parenting Efficacy Scale (PES): This 10-item PES scale [41] is used to measure parental beliefs on self-efficacy. Nine of the items refer to specific behaviors related to infant care (eg, feeding and bathing), and the last item is a global evaluation of parenting ability. The total score of PES ranges from 10 to 40, with higher scores indicating higher PSE. The Cronbach alpha value of the PES was .74 in the previous study [42].
2. Parent- Infant Bonding Questionnaire (PIBQ): This is an 8-item, 4-point Likert scale used to measure the parental bonding of both parents [43]. The total scores range from 0 to 24, with higher scores suggesting ineffective bonding between mother and infant and vice versa. The cut-off score indicating poor bonding between parent and infant is indicated at 2 [44]. The Cronbach alpha for this questionnaire was .7 in the previous study [45].
3. Edinburgh Postnatal Depression Scale (EPDS): This is a 10-item scale widely used to measure parents’ postnatal depression [24,29,46-48]. The total score of the EPDS ranges from 0 to 30. The recommended cut-off score of 12 or 13 is used for screening probable cases of postnatal depression. The sensitivity of the EPDS ranges from 68% to 80%, with 77% specificity and a Cronbach alpha of .88 [49].
4. State Trait Anxiety Inventory (STAI): This is a 40-item 4-point Likert scale used to measure parental anxiety level. The total score for the STAI ranges from 40 to 160, with higher scores indicating greater levels of parental anxiety. The scale has been tested widely in various studies [50-53]. The Cronbach alpha for the STAI was .8 in a previous local study [52].
5. Perceived Social Support for Parenting (PSSP): This is a 4-item scale [42] used to measure the social support parents receive from their partners or others. The score of the 5-point Likert scale ranges from 0 to 20 for the support received from partners or others. The instrument showed high internal consistency and had a Cronbach alpha of .81 in a previous study [42].
6. What Being the Parent of a Baby is Like (WBPL): The evaluation subscale of the WBPL will be used to measure parenting satisfaction [54]. It consists of 11 items, and each item is a 10-point semantic differential scale ranging from 0 (none of the phenomenon) to 9 (a great deal of the phenomenon). In previous studies, the Cronbach Alpha ranged from .91 to .92 [54,55].
7. Health care utilization and program-related expenses sheet: This sheet will be used to capture all direct health systems cost related to health care services utilized antenatally and postnatally because of maternal or infant related health issues such as hospital admissions and outpatient contacts, health professional’s time, program-related time, and during pregnancy, for the first and 3rd month after delivery. The study will take an *all-payer* health system perspective in which it will attempt to capture all resources that are used by the participants, regardless of who the payer is (ie, government or consumer), including the program cost. The measurement period begins from entry into the program until 3 months post birth.
8. A semistructured interview guide will be used for the process evaluation. Individual face-to-face interviews will be conducted immediately after the intervention (1 month post birth) to identify strengths and weaknesses and to comment on the content, activities, effectiveness, and delivery methods of the SEPP for participants in the intervention. Parents in the control group will be asked to comment on the current routine perinatal care provided by the hospital. All interviews will be audiorecorded.

### Study Procedure

The study consists of two phases: (1) planning intervention strategies for the intervention group, including the development of the educational content to be covered during the antenatal and postnatal periods and the development of the mHealth app, audio, and videos related to postnatal care, and (2) implementation of the SEPP and investigating its effectiveness on parental outcomes.

This study has been approved by the ethics board, and recruitment at the antenatal clinics has started at the study venue. The principle investigator and her team will inform nurse managers and clinicians at the antenatal clinics before a RA (RA1) commences the process of recruitment. Before contacting potential participants, the attending nurses and/or clinicians will be approached to assess each couple’s physical and psychological well-being before inviting them to participate in the research study. The referred couples will once again be assessed using the inclusion and exclusion criteria. There will be no undue influence or coercion used to recruit couples into the study. Each couple will be given details of the purpose of the study and ample time to ask questions and to consider before deciding to participate. It will be strongly emphasized that their participation is solely voluntary and that they may choose not to participate without any harm or compromise in the care they receive. Verbal and written consent will be obtained from the couple, both mother and father, thereafter. Both first-time and experienced mothers, regardless of their parity, who meet the inclusion criteria, along with their partners, will be recruited. RA1 will visit the antenatal clinics regularly to recruit participants according to the inclusion and exclusion criteria and will be responsible for randomizing the participants.

Once consent has been taken, demographic and baseline data of the participants will be obtained, and randomization will take place. In addition, recruited couples will be asked for their contact details so that they can be contacted for subsequent follow-ups. RA1 will keep in constant contact with the participants to ensure the birth of the newborn. For participants in the intervention group, telephone-based antenatal education session will be delivered by RA1 at the participants’ convenience at a prearranged timeslot.

After childbirth, all couples will be reapproached in the postnatal wards by the RA before their day of discharge post childbirth. Before making contact in the postnatal wards, the nurse-in-charge will be approached by RA1 to ensure the physical and emotional well-being of the participants. For participants in the intervention group, the postnatal education session will be delivered by RA1 in the postnatal wards on the day of discharge from the hospital. RA1 will assist parents in downloading the mHealth app and demonstrate the functions of the app before discharge. Participants will also be given individual usernames and passwords for access to the mHealth app. Access to the app will expire automatically in 4 weeks. RA1 will be responsible for answering the queries in the discussion forum and to monitor and correct any inappropriate content shared among parents.

### Data Collection

**Figure 7:**
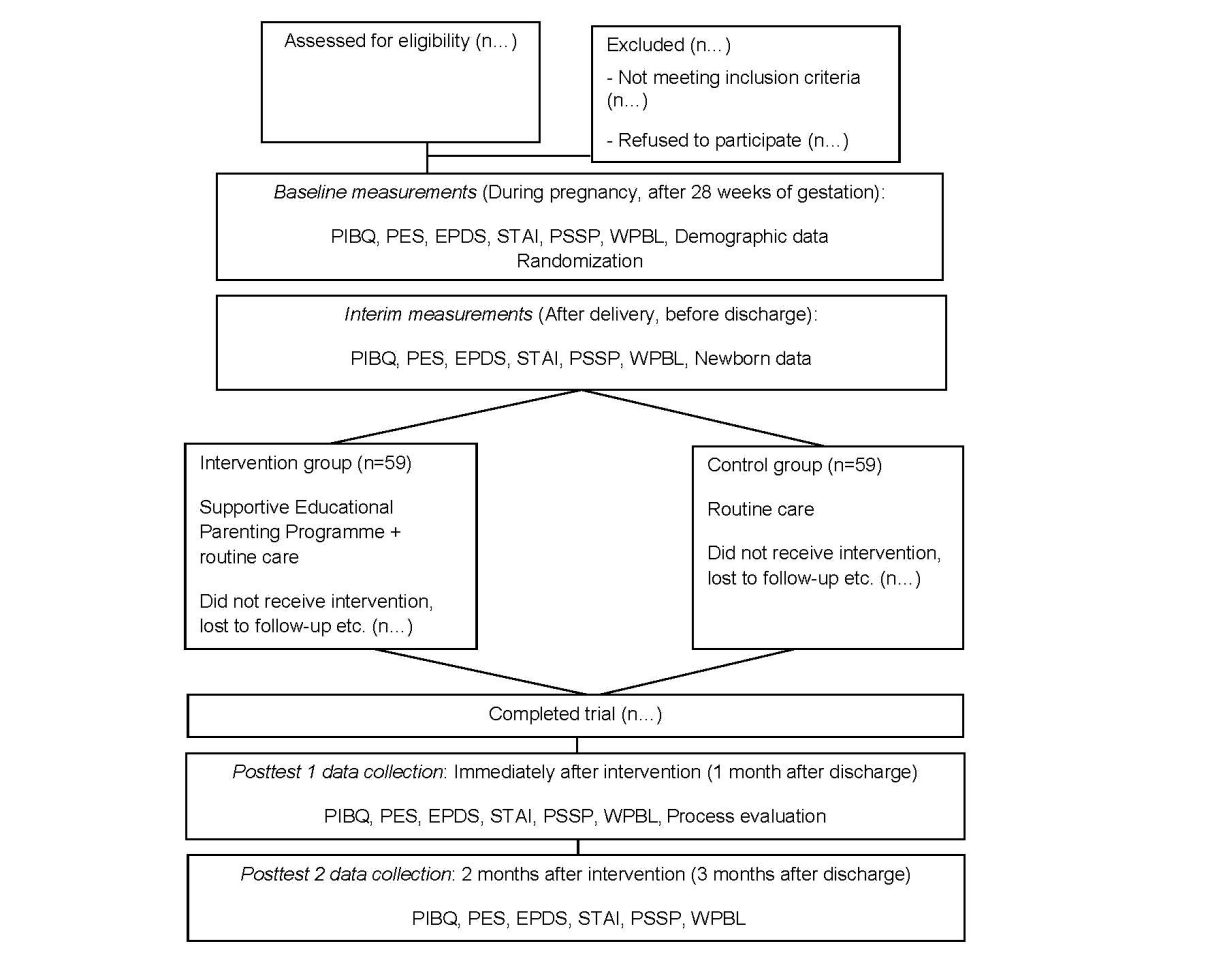
The consolidated standards of reporting trial flowchart. PIBQ: Parent-Infant Bonding Questionnaire; PES: Parenting Efficacy Scale; EPDS: Edinburg Postnatal Depression Scale; STAI: State-Trait Anxiety Inventory; PSSP: Perceived Social Support for Parenting; and WBPL: What Being the Parent of a Baby is Like.

A single-blind technique will be adopted, in which the RA (RA2) responsible for the collection of data post intervention or post discharge will not be aware of the treatment allocation, which will be conducted by another RA (RA1). RA2 will be responsible for the collection of all interim and postintervention data, including the process evaluation interviews. Outcome measures will take place at the following time points for all parents: (1) during pregnancy, before randomization (baseline); (2) after childbirth, before hospital discharge (interim data); (3) post intervention for the SEPP intervention group and at the end of week 4 post childbirth for the control group (posttest 1); and (4) 2 months after the SEPP for the intervention group and 3 months post childbirth for the control group (posttest 2). The period when parents are prone to facing various challenges is at 1 month post birth. Hence, the rationale for collecting posttest 1 data during that time. At 3 months post birth is when some working mothers return to work, signifying a change in environment relating to the rational for collecting posttest 2 data. Before contacting parents for the posttest data, a message will be sent to ensure parents’ availability. In the process of data collection, if either parent feels tired, RA2 will respect the parents’ decision to continue or terminate the data collection to ensure parents’ comfort. The consolidated standards of the reporting trial flowchart for this study is presented in Figure 7 [56].

### Process Evaluation

Process evaluation will be conducted with the main objective of obtaining parents’ opinions and comments of the SEPP intervention. Purposive sampling of parents will be done based on the parenting self-efficacy scores of parents immediately after the end of the intervention (at 1 month post childbirth). This will be done through a semistructured face-to-face interview. Each interview will take approximately 20 to 30 min and will be audiorecorded. Parents will not be included for interview if they refused to be audiorecorded. The recruitment for process evaluation will continue and will only be terminated once the proposed number of interviews is reached or when data saturation is achieved. The semistructured interview guide has been reviewed and approved by the ethics board.

### Data Analysis

All quantitative data will be analyzed using Statistical Package for the Social Sciences (SPSS) version 24.0 (IBM Corp). Missing data will be replaced (assuming 10%) for intention-to-treat (ITT) analysis. Both ITT analysis and per-protocol analysis will be conducted to compare any differences between groups. Descriptive statistics such as mean, standard deviation, and range for continuous dates, frequencies, and percentages will be used for nominal and ordinal data. Cronbach alpha will be used to test the internal consistencies of the questionnaires. Inferential statistics such as the independent *t* test or ANOVA will be used to compare differences of outcomes between or among demographic subgroups. Presuming that outcomes variables are normally distributed, parametric tests will be used. Repeated measures ANCOVA adjusted for confounding variables will be used to test the effect of intervention on outcome variables across four time points. Univariate ANCOVA will be used to test the differences in outcome variables between two groups at the interim and two posttests data separately.

Qualitative data will be analyzed using thematic analysis [57,58]. All recorded audio will be transcribed verbatim by the RA2 concurrently with data collection to capture nonverbal information. Transcribed data will be classified into different categories, with similar ideas grouped in a category highlighted in the same color. Related codes or categories will be collated together to form subthemes, which will be reviewed and combined to form themes [57]. Two investigators will be involved in the analysis process to compare and discuss the categories, subthemes, and themes that are generated and to achieve consensus. Rigor or trustworthiness, including credibility, transferability, dependability, and confirmability, will be considered carefully [58].

### Ethical Considerations

Ethics approval has been obtained from the National Health Group Domain Specific Review Board (NHG DSRB) before the commencement of the study (Ref. No: NHG DSRB: 2016/00651) in June 2016. All participants are given a set of participant information sheets consisting a brief introduction and the purpose of the study, with the advantages and disadvantages of the study conveyed clearly. The participants are guaranteed anonymity and are informed of the right to withdraw at any point of the study without affecting any subsequent care received.

## Results

Phase 1 of the study has been completed. Educational materials for both antenatal and postnatal education, audio and videos files, and the mHealth app have been developed. For phase 2, the recruitment of study participants commenced in December 2016 and is still ongoing. The targeted aim of recruiting 118 couples will be ongoing for a period of 12 months. The total number of participants approached thus far are 211, and 162 were screened (not interested and did not comply to one of the inclusion criteria). The current enrollment is 49 couples (for both the intervention [n=27] and control [n=22] groups). The projected timelines for the completion of the data entry and analysis for investigating the SEPP’s effectiveness on parental outcomes is around December 2017.

## Discussion

Theoretical framework–based and technology-enhanced educational programs have shown effectiveness [22,24,25,30,31] in influencing parental outcomes in the postnatal period. Previous studies have used varied technologies including telephone calls [31], Web-based programs [30], and mHealth apps [22,25] to influence postnatal depression [31], parenting satisfaction, and self-efficacy [30]. The only local study [25] that has used an mHealth app in supporting parents has influenced parental outcomes. However, the effectiveness was tested short-term (1 month post childbirth), and the study showed no influence on parental postnatal depression. A few studies [22,24,25,30,31] recommended the need of providing long-term perinatal educational support to parents. Specifically, the previous local study [25] recommended a long-term evaluation of the effectiveness of the educational program beyond 1 month. As such, this study will address this gap in literature by providing empirical support for the feasibility and effectiveness of the SEPP in enhancing parental outcomes across the perinatal period. This will also be the first study in Singapore that will examine the cost-effectiveness of an educational intervention delivered to parents.

We are hopeful that the provision of this technology-based educational program, which includes telephone calls and an mHealth app, will provide a clinically useful and potentially cost-effective solution to improve parental outcomes. This may eventually lead to more positive perinatal parenting experiences, which may influence the psychosocial well-being of both parents and their newborn, leading to better family dynamics and reducing associated health care and social burdens. It may also impact the future reproductive decisions of parents.

It is worthy to note that this study is being tested in a multiracial population, which enhances its relevance in the local context as participants in the study venue have similar demographic profile to Singapore’s general population, with the majority of its population being Chinese, followed by Malays and Indians. However, being a single-centered study, it limits its generalizability to an international population and requires further evaluation.

## Abbreviations

ANCOVA: analysis of covariance
EPDS: Edinburg Postnatal Depression Scale
HCP: health care professional
ITT: intention to treat
mHealth: mobile health
PES: Parenting Efficacy Scale
PIBQ: Parent-Infant Bonding Questionnaire
PSE: parenting self-efficacy
PSSP: perceived social support for parenting
RA: research assistant
SEPP: Supportive Educational Parenting Program
STAI: State Trait Anxiety Inventory
WBPL: What Being the Parent of a Baby is Like
